# Effects of liquid stimuli on dual-axis swallowing accelerometry signals in a healthy population

**DOI:** 10.1186/1475-925X-9-7

**Published:** 2010-02-04

**Authors:** Joon Lee, Ervin Sejdić, Catriona M Steele, Tom Chau

**Affiliations:** 1 Bloorview Research Institute, 150 Kilgour Road, Toronto, Ontario, Canada; 2 Toronto Rehabilitation Institute, 550 University Avenue, Toronto, Ontario, Canada; 3 Department of Electrical and Computer Engineering, University of Toronto, Toronto, Ontario, Canada; 4 Institute of Biomaterials and Biomedical Engineering, University of Toronto, Toronto, Ontario, Canada; 5 Department of Speech-Language Pathology, University of Toronto, Toronto, Ontario, Canada

## Abstract

**Background:**

Dual-axis swallowing accelerometry has recently been proposed as a tool for non-invasive analysis of swallowing function. Although swallowing is known to be physiologically modifiable by the type of food or liquid (i.e., stimuli), the effects of stimuli on dual-axis accelerometry signals have never been thoroughly investigated. Thus, the objective of this study was to investigate stimulus effects on dual-axis accelerometry signal characteristics. Signals were acquired from 17 healthy participants while swallowing 4 different stimuli: water, nectar-thick and honey-thick apple juices, and a thin-liquid barium suspension. Two swallowing tasks were examined: discrete and sequential. A variety of features were extracted in the time and time-frequency domains after swallow segmentation and pre-processing. A separate Friedman test was conducted for each feature and for each swallowing task.

**Results:**

Significant main stimulus effects were found on 6 out of 30 features for the discrete task and on 5 out of 30 features for the sequential task. Analysis of the features with significant stimulus effects suggested that the changes in the signals revealed slower and more pronounced swallowing patterns with increasing bolus viscosity.

**Conclusions:**

We conclude that stimulus type does affect specific characteristics of dual-axis swallowing accelerometry signals, suggesting that associated clinical screening protocols may need to be stimulus specific.

## Background

Dysphagia refers in general to swallowing disorders [1], and is a common consequence of neurological conditions such as stroke, cerebral palsy, or Parkinson's disease [2]. Adverse effects of dysphagia include degraded psycho-social well-being [3], dehydration and malnutrition [4,5], and compromised immune system secondary to malnutrition [4]. Furthermore, dysphagia can jeopardize airway protection during pharyngeal swallowing, heightening the risk of entry of foreign material into the unprotected airway during swallowing (aspiration), which may lead to aspiration pneumonia [6]. Devastating outcomes of aspiration pneumonia range from hospitalization to death [7]. The current gold standard in dysphagia assessment is the videofluoroscopic swallowing study (VFSS) [1,8]. In this imaging technique, X-ray video of the pharyngeal region is recorded while the patient swallows food or liquid stimuli mixed with barium. The primary objectives of VFSS are to determine the nature and severity of dysphagia and to devise appropriate intervention techniques. Many smaller healthcare institutions are unable to provide VFSS, resulting in long wait times for patients with dysphagia [9]. In addition, VFSS is neither practical nor feasible for long-term or day-to-day monitoring of dysphagia.

Recognizing the limitations of VFSS access, several alternative techniques have been investigated. Examples include pulse oximetry [10], cervical auscultation [11], and electrophysiological methods [12]. All these techniques employ non-invasive signal modalities and easy-to-attach sensors. Among such alternatives, swallowing accelerometry is the cervical vibration measurement technique that utilizes an accelerometer, and has been the focus of several recent studies (e.g. [13,14]). Notably, a few of these studies have incorporated digital signal processing and pattern recognition schemes in an effort to automatically detect abnormal swallows [13,15]. Successful diagnostic algorithms based on swallowing accelerometry have the potential to be implemented as a portable device that might be used for screening or day-to-day monitoring. Although single-axis accelerometry in the anterior-posterior (A-P) direction has been extensively studied, a recent dual-axis accelerometry study showed that the superior-inferior (S-I) direction contains swallowing information that is absent in the A-P direction [14].

The physiological origins of dual-axis accelerometry signals are yet to be completely elucidated. However, given the cervical location of sensor placement, the dominant source is presumably the mechanical movement of the hyolaryngeal structure during the oral and pharyngeal phases of swallowing. It is logical to expect that the esophageal phase would generate negligible vibrations that can be measured at the thyroid cartillage following the descent of the hyoid and larynx at the conclusion of the pharyngeal phase. This has recently been supported by Zoratto *et al*. [16], whose study showed that the hyoid movement during swallowing at least partially contributes to dual-axis accelerometry signals. Also, Reddy *et al*. [17] reported that there exists a significant correlation between the A-P accelerometry signal and the the extent of laryngeal elevation during swallowing. An acoustic source has been proposed as well [18], but was rejected as a meaningful source of dual-axis accelerometry [14].

Although deglutition (i.e. swallowing) involves a well-defined pattern of neuronal activations, it can be modified according to sensory information conveyed from the oral cavity and pharynx [2,19]. Afferent neurons associated with mechanoreceptors and chemoreceptors are largely responsible for such sensory feedback, and they are primarily influenced by stimulus characteristics. The use of thickened liquids is a well-known clinical application of swallow modification using different stimuli to elicit safer and more efficient swallows [20,21].

A number of studies have investigated how stimulus characteristics, including viscosity, density, volume, taste, and texture, influence swallowing behaviours. Steele *et al*. [20] considered thin, nectar-thick, and honey-thick liquids and found that viscosity and density influenced the durations of oropharyngeal transit and downward tongue dorsum movement, measured using electromagnetic midsagittal articulography. However, they found no other stimulus effects on tongue behaviors in healthy adults. Logemann *et al*. [22] showed beneficial effects of sour taste on several swallowing measures in patients with dysphagia. In a similar study, Pelletier and Lawless [21] reported that airway invasion was significantly mitigated in patients with neurogenic oropharyngeal dysphagia by using a high-intensity sour stimulus (citric acid) as opposed to water. They speculated that the observed improvement in swallowing could be attributable to enhanced gustatory and trigeminal stimulation. Moreover, Chi-Fishman and Sonies [23] used ultrasonography to study the effects of bolus viscosity and volume on hyoid kinematics in healthy individuals. They found significant main effects of viscosity on the duration of hyoid movement and of volume on the extent of hyoid excursion and hyoid movement velocity. Lastly, the principle that stimulus characteristics might influence swallowing is supported by a monkey study by Martin *et al*. [24], which showed that the majority of the swallow-related neurons in the tongue primary motor cortex were associated with a mechanoreceptive field on the tongue dorsum.

In spite of the known effects of stimulus characteristics on swallowing function, there is a lack of knowledge to date regarding stimulus effects on dual-axis accelerometry signals. This is a particularly important issue for abnormal swallow detection based on accelerometry, because observed differences between healthy and abnormal swallows might in fact reflect normal variations attributable to the influences of different stimuli. Furthermore, this question needs to be addressed for the development of an abnormal swallow detection device, so that either the use of the device can be limited to one particular stimulus type or the detection algorithm is designed to be stimulus-independent or stimulus-adaptive. Although our previous study found no significant stimulus effects on a small set of features extracted from dual-axis accelerometry signals in a healthy population [14], a more comprehensive investigation is needed to provide a definitive answer. Dependence on stimuli has been identified in other swallowing-related signals such as electromyography [25,26] and nasal airflow [27].

The main contribution of the present study is the investigation of the effects of four different liquid stimuli on a broad collection of time and time-frequency features extracted from dual-axis swallowing accelerometry signals, acquired from healthy individuals. The analysis is strictly from a signal standpoint, since the relationship between swallowing physiology and dual-axis accelerometry is still unclear as mentioned above. The results of this study will provide new insight into the ways in which modulations of swallowing function are manifested in accelerometry signals. We anticipate that the results of this study would be of interest to clinicians interpreting accelerometry signals for dysphagia assessment and to engineers developing diagnostic algorithms based on accelerometry.

## Methods

### Signal Acquisition

Seventeen healthy adults (8 males) with no history of dysphagia or any neurological impairment participated in this study. Prior to acceptance into the study, all participants underwent a standardized oral mechanism examination and a water swallow screening test conducted by a registered speech-language pathologist to confirm the absence of any clinical signs of dysphagia. The mean age of the participants was 46.9 ± 23.8 years. This study was approved by the research ethics committees at the Toronto Rehabilitation Institute, Bloorview Kids Rehab, and University of Toronto. Each participant gave informed consent prior to participation.

Each participant was asked to swallow 4 different liquid stimuli: water, a 40% weight per volume thin liquid barium suspension (prepared using water and Liquid Polibar™ barium, E-Z-EM), and commercially prepared pre-thickened nectar-thick and honey-thick apple juices (RESOURCE^®^, Novartis Nutrition). All stimuli were self-administered via a cup. No standardized sip volume was enforced, and the stimuli were served chilled. For each stimulus, each participant completed sequences of 4 swallows in two different tasks: discrete and sequential swallowing. In the discrete task, participants were instructed to take 4 sips of a comfortable size and to remove the cup from the lips between consecutive sips. In the sequential task, participants were asked to take 4 consecutive sips without taking the cup off the lips. For both tasks, it was made clear that there was no requirement to finish the contents of the cup for each sequence. Participants were also asked to initiate each swallowing sequence upon hearing an audible cue, which was generated 5 seconds after signal recording started. Participants were allowed to complete each swallowing sequence at a self-selected, comfortable pace. Each stimulus-task combination was repeated twice, with the exception of the water-discrete combination, which was repeated 3 times, yielding 17 sequences per participant in total.

**Figure 1:**
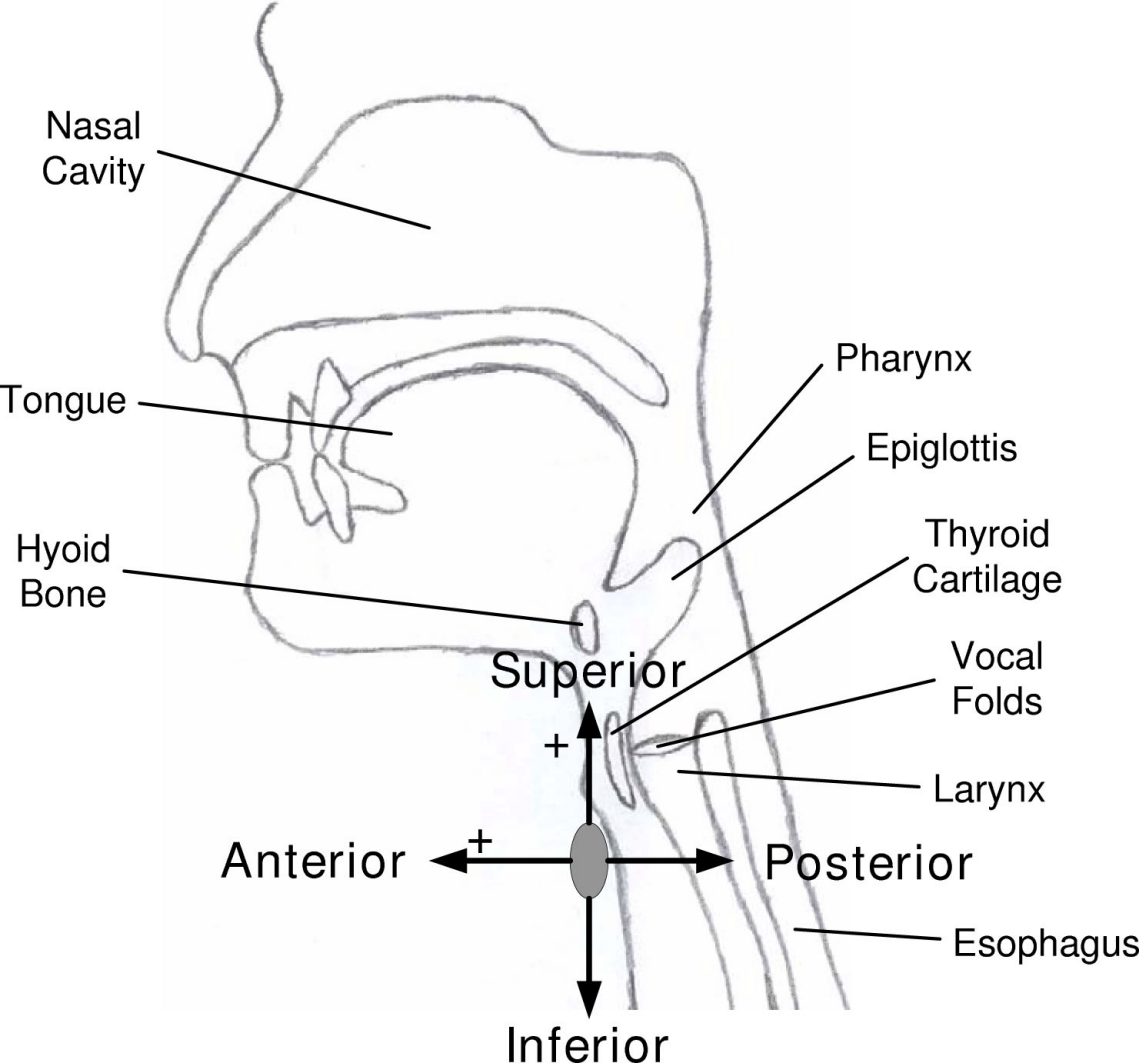
**Accelerometry sensor placement**. A sagittal view of the cervical region showing the orientation and polarity of the two axes of the accelerometer and nearby anatomical structures.

Dual-axis accelerometry signals were acquired via an accelerometer (ADXL322, Analog Devices) placed at a midline cervical location just below the thyroid cartilage. The attachment was accomplished with a double-sided electrode collar (650455, VIASYS Healthcare). The two axes of the accelerometer were oriented along the A-P and S-I directions. See Figure 1 for an illustration of the sensor placement and axial orientation. Each of the two channels corresponding to the two axes was then passed through a pre-amplifier with a bandpass filter (Model P55, Grass Technologies, West Warwick, RI). The amplifier provided 10-times amplification, and the lower and upper cutoff frequencies of the bandpass filter were set at 0.1 Hz and 3 kHz, respectively. Both signal channels were sampled by a custom LabVIEW application at 10 kHz. These filtering, amplification, and sampling specifications have been justified in a previous swallow accelerometry study [14].

### Swallow Segmentation and Pre-processing

The 4 swallows in each sequence were segmented using the sequential fuzzy c-means algorithm described in [28], which was designed for dual-axis accelerometry signals. Subsequently, the results of this automatic segmentation were visually verified, and any incorrectly segmented swallows were manually segmented based on visual inspection. However, some swallows could not be segmented even by the manual segmentation and hence were excluded from the study. In the end, 1,114 swallows were available for pre-processing and analysis.

Each segmented signal was then filtered through axis-specific finite impulse response (FIR) filters that were designed to annul the unwanted effects introduced by the signal acquisition system [29]. The modified covariance method of autoregressive (AR) modeling was utilized to model the data acquisition system using baseline recordings, and inverse filtering on the constructed AR model yielded the FIR filters. Next, the segmented and filtered signals were denoised via a 10-level discrete wavelet decomposition with the discrete Meyer wavelet and soft-thresholding. The global denoising threshold, *T*_*den*_, was determined empirically based on the 1st-level detail signal, *d*_1_, as follows [30]:(1)

where *n *is signal length and *med *is the median operator.

### Feature Extraction

For each swallow, a number of time and time-frequency features were extracted from the pre-processed A-P signal, *X *= {*x*_1_, *x*_2_, ..., *x*_*n*_}, and S-I signal, *Y *= {*y*_1_, *y*_2_, ..., *y*_*n*_}. A variety of features were deployed to obtain a comprehensive description of the dual-axis accelerometry signals corresponding to each swallow, from a signal perspective. The following subsections describe the computation of each feature in detail. For the features that were identically extracted from both axes, the description below is based only on the A-P signal, *X*. The same formulation applies for the S-I signal.

#### Time Domain Features

• The mean of the amplitude values of the signal is a measure of the location of the amplitude distribution, and was computed as follows:(2)

• The variance of the amplitude distribution of the signal is a measure of the spread of the distribution as well as AC signal power. An unbiased estimate of the variance was computed as follows:(3)

• The skewness of the amplitude distribution is a measure of the asymmetry of the distribution, and was computed as follows:(4)

Skewness has been employed in characterization of the dual-axis accelerometry signal [14].

• The kurtosis of the amplitude distribution is a measure of the peakedness of the distribution, and was computed as follows:(5)

This higher moment has been utilized in a previous dual-axis accelerometry study [14].

• The entropy rate measure introduced by Porta *et al*. [31,32] quantifies the extent of regularity in a signal and is particularly useful for characterizing a stochastic process in which some relationship among consecutive data points is anticipated. These kinds of relationships are expected in swallowing accelerometry given that swallowing is a well-defined physiological process. Prior to the actual computation of the entropy rate feature, *X *was normalized to zero mean and unit variance, by subtracting *μ*_*X *_and dividing by *σ*_*X*_. The normalized *X *was then quantized into 10 equally spaced levels represented by integers from 0 to 9, ranging from the minimum to maximum value. With the quantized signal denoted as , sequences of consecutive points in  of length *L*, 10 ≤ *L *≤ 30, *L *∈ ℤ^+^, were coded as a series of integers, Ω_*L *_= {*w*_1_, *w*_2_, ..., *w*_*n*-*L*+1_}, according to the following:(6)

This implies that *w*_*i *_ranged from 0 to 10^*L*^-1. Base 10 was used because there were 10 quantization levels. The Shannon entropy of Ω_*L *_was defined as follows:(7)

where  (*j*) represents the probability of the value *j *in Ω_*L*_, approximated by the corresponding sample frequency. The normalized entropy rate was then computed as follows:(8)

where *perc*(*L*) is the percentage of the coded integers in Ω_*L *_that occurred only once. Finally, an index of regularity, *ρ*, was calculated as the entropy rate feature in this study:(9)

which ranged from 0 (maximum randomness) to 1 (maximum regularity).

• The memory of a signal quantifies the temporal extent of the correlation among nearby data points. To compute a measure of signal memory, the autocorrelation of the signal was computed from zero to the maximum possible time lag and was normalized so that the autocorrelation at zero lag was unity. The memory was then estimated by the 1/*e *width, which is the time duration from zero lag to the point where autocorrelation becomes less than or equal to 1/*e *≈ 0.3679. This particular threshold has been used before to estimate signal memory in dual-axis accelerometry [14].

• The Lempel-Ziv (L-Z) complexity [33] is a measure of the predictability of the signal. It has been applied in a number of biomedical applications, ranging from complexity characterization of DNA sequences [34] to analyses of brain information transmission [35]. Further, Aboy *et al*. [36] discussed how to interpret the L-Z complexity in the realm of biomedical signal analysis and concluded that the L-Z complexity is a useful scalar estimator of the bandwidth of a random process and the harmonic variability in quasi-periodic signals. Prior to the computation of the actual complexity value, *X *was first converted to a sequence of finite symbols by using 100 equally spaced quantization levels ranging from the minimum to the maximum in *X*. Next, the quantized signal  = {*s*_1_, *s*_2_, ..., *s*_*n*_} was decomposed into *k *blocks so that  = [Φ_1_, Φ_2_, ..., Φ_*k*_]. A block was a sequence of consecutive symbols of length ℓ - *j *+1 expressed as follows:(10)

The first block was simply initialized to be the first symbol, i.e. Φ_1 _=  = *s*_1_. Subsequent blocks were determined to be(11)

where *h*_*m *_is the ending index for Φ_*m*_, such that Φ_*m*+1 _is a unique sequence of minimal length in the sequence . Finally, the normalized complexity was computed as follows and utilized in this study:(12)

The logarithmic base of 100 came from the fact that there were 100 quantization levels.

• The temporal swallow duration in seconds, 0.0001(*n-*1), was utilized as a feature. Bolus consistency has been shown to influence the duration of the pharyngeal swallow [2,20,23].

• Extending from the entropy rate measure above, Porta *et al*. [37,38] also introduced a method of quantifying the cross-entropy rate between two stochastic processes. This measure describes the predictability of a data point in one signal given a sequence of current and past data points in the other signal. First, both *X *and *Y *were normalized, quantized, and coded using the same methodology as for the entropy rate feature, yielding  and , respectively, with 10 ≤ *L *≤ 30, *L *∈ ℤ^+^. In addition,  was constructed as follows:(13)

where  and  are the quantized signals. Then, with *SE*_*X *_(*L*), *SE*_*Y*_(*L*), and *SE*_*X*/*Y *_(*L*) representing the Shannon entropies (defined in (7)) of , , and , respectively, the normalized cross-entropy of *X *given *Y *was computed as follows:(14)

where *perc*_*X*/*Y *_(*L*) is the percentage of the elements in  that occurred only once. Next, the uncoupling function was defined as follows:(15)

Finally, the following index of synchronization was computed and utilized as the cross-entropy rate feature in this study:(16)

which ranged from 0 (*X *and *Y *are completely uncoupled) to 1 (perfect synchronization).

• The cross-correlation between *X *and *Y *at zero lag, *R*_*XY *_(0), was computed as follows:(17)

Liao [39] provides a review of cross-correlation as a useful measure of dissimilarity between two time series.

#### Time-frequency Domain Features

• The relative energy in each wavelet decomposition level was extracted from a 10-level discrete wavelet decomposition of the signal using the discrete Meyer wavelet. Such wavelet energy features have been widely applied (e.g. [14,40-42]). If the decomposition is denoted as *W*_*X *_= [*a*_10 _*d*_10 _*d*_9 _... *d*_1_], where *a*_10 _is the approximation signal and *d*_*k *_is the *k*^*th*^-level detail signal, the energy in the approximation signal was computed as follows:(18)

where ||•|| is the Euclidean norm. Similarly, the energy in the *k*^*th*^-level detail signal was:(19)

for *k *= 1, 2, ..., 10. Finally, the relative energy contribution from each decomposition level was computed as follows:(20)

for *k *= 1, 2, ..., 10 and where(22)

**Figure 2:**
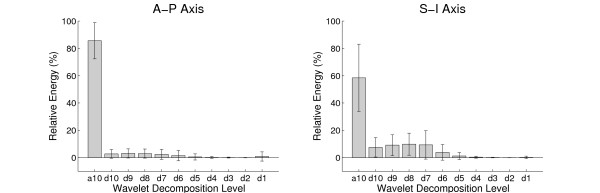
**Axial energy contribution from wavelet decomposition**. Relative energy contribution from each wavelet decomposition level in each axis, averaged over all swallows from both discrete and sequential tasks. Vertical and error bars represent means and standard deviations, respectively.

Figure 2 shows a bar graph for each axis that visually illustrates the extracted energy features, averaged over all swallows from both swallowing tasks. It is clear in this figure that several decomposition levels in high frequencies contributed negligible energy. Hence, only the energy features from the lower spectral bands with meaningful signal energy were employed as features in this study. The cutoff was determined by accumulating mean energies from the lowest spectral band, *a*_10_, until 95% of total energy was reached. All the remaining decomposition levels were excluded from further statistical analysis. As a result, the relative energy features for the levels from *d*_1 _to *d*_6 _and from *d*_1 _to *d*_5 _were discarded for the A-P and S-I axes, respectively.

• A wavelet entropy measure based on contributions from different wavelet decomposition levels was utilized as a feature [40,43]. The definition of this feature requires all decomposition levels, including the discarded levels in the computation of the relative energy features. Using the same 10-level wavelet decomposition and relative energies computed above for the wavelet energy features, the wavelet entropy was computed as follows:(23)

### Statistical Tests

Main stimulus effects were examined with non-parametric Friedman tests at a significance level of *α *= 0.05. Stimulus and participant were the two independent variables in the tests, but we only focused on main stimulus effects. A separate Friedman test was conducted for each of the two swallowing tasks and for each feature as the dependent variable. In order to compensate for differences in the number of swallows for different stimulus-participant combinations arising from variable segmentation results, the median in each stimulus-participant combination was used in the Friedman tests. The robustness of the median statistic ensured that outliers had minimal impact on the outcomes of the Friedman tests.

For the features with significant main effects of stimuli, post-hoc pairwise comparisons tests were conducted using Wilcoxon ranksum tests with the Bonferroni correction at a familywise significance level of *α *= 0.05.

## Results and Discussion

Tables 1 and 2 tabulate statistically significant results from the Friedman tests for the discrete and sequential tasks, respectively. Of the 30 features considered, 6 and 5 features exhibited significant main stimulus effects for the discrete and sequential tasks, respectively. This implies that the majority of the features considered in the present study showed no stimulus effects. In terms of the post-hoc analysis for the discrete task, only the pairwise comparison between honey-thick and barium for entropy rate S-I resulted in a significant difference (*p *= 0.008). For the sequential task, significant post-hoc results were found for entropy rate A-P, between nectar-thick and barium (*p *= 0.0055), and honey-thick and barium (*p *= 0.0055), as well as for entropy rate S-I, between water and honey-thick (*p *= 0.0079), nectar-thick and barium (*p *= 0.0028), and honey-thick and barium (*p *= 0.0008). Based on the fact that entropy rate and L-Z complexity based features were significantly influenced by stimuli, it is inferred that stimuli modified the predictability and regularity of the dual-axis accelerometry signals. Furthermore, the significant post-hoc results for entropy rate as well as the corresponding mean values in Tables 1 and 2 provide evidence that signal regularity was higher for stimuli with higher viscosity (water *<*barium *<*nectar-thick *<*honey-thick, in terms of viscosity). Also, the mean L-Z complexity measures in Tables 1 and 2 generally show a similar dependence on viscosity. These findings indicate that the accelerometry signals exhibited a more prominent, well-defined pattern as bolus viscosity increased, which resonates with the clinical observation that swallowing function tends to be reinforced and stronger with thicker liquids [20,21]. However, it is worthwhile to note that the numerical differences among stimuli for the entropy rate and L-Z complexity features in Tables 1 and 2 are small given that the two features were normalized to range from 0 to 1.

**Table 1:** Features with significant main stimulus effects: discrete swallows (mean ± standard error)

	Stimulus				
		
Feature	Water	Barium	Nectar-thick Apple Juice	Honey-thick Apple Juice	*p*-value
Skewness A-P	-1.171 ± 0.246	-1.190 ± 0.258	-0.893 ± 0.256	-0.762 ± 0.207	0.0416
Entropy rate A-P	0.982 ± 0.002	0.980 ± 0.002	0.985 ± 0.002	0.987 ± 0.002	0.0009
Entropy rate S-I	0.986 ± 0.002	0.986 ± 0.001	0.988 ± 0.002	0.989 ± 0.001	0.0060
L-Z complexity A-P	0.071 ± 0.004	0.070 ± 0.004	0.063 ± 0.004	0.060 ± 0.004	0.0212
L-Z complexity S-I	0.084 ± 0.004	0.087 ± 0.005	0.079 ± 0.005	0.076 ± 0.005	0.0403
Duration (s)	2.097 ± 0.077	2.056 ± 0.074	2.228 ± 0.083	2.324 ± 0.094	0.0018

**Table 2:** Features with significant main stimulus effects: sequential swallows (mean ± standard error)

	Stimulus				
		
Feature	Water	Barium	Nectar-thick Apple Juice	Honey-thick Apple Juice	*p*-value
Entropy rate A-P	0.979 ± 0.002	0.979 ± 0.002	0.985 ± 0.001	0.986 ± 0.001	0.0040
Entropy rate S-I	0.983 ± 0.002	0.982 ± 0.002	0.987 ± 0.001	0.988 ± 0.001	*<*0.0001
L-Z complexity S-I	0.103 ± 0.006	0.108 ± 0.007	0.091 ± 0.005	0.094 ± 0.005	0.0391
Duration (s)	1.463 ± 0.081	1.533 ± 0.072	1.643 ± 0.063	1.747 ± 0.105	0.0105
Wavelet energy *d*_9 _S-I (%)	7.814 ± 1.437	9.254 ± 1.529	9.816 ± 1.977	9.327 ± 1.065	0.0105

Swallow duration was also significantly affected by stimuli during both swallowing tasks. Tables 1 and 2 show that nectar-thick and honey-thick apple juices were associated with longer swallow durations on average than water and barium. This corroborates previous findings that several physiological components of swallowing are delayed or prolonged by more viscous boluses, as measured by electromyography [44] and videofluoroscopy/manometry [45]. Specifically, increasing bolus viscosity is known to be associated with delay in oral and pharyngeal bolus transit, a longer period of pharyngeal peristaltic waves, and prolonged upper esophageal sphincter opening.

As shown in Table 1, nectar-thick and honey-thick apple juices yielded less negatively skewed A-P amplitude distributions than water and the barium suspension. Negative skewness indicates that the mass of the distribution is concentrated on the right side. Because positive amplitude corresponded to the anterior direction (see Figure 1), we speculate that acceleration in the anterior direction or deceleration in the posterior direction was more prominent when water or barium was swallowed than when either of the two apple juices was taken.

The results of this study differ from those of our previous study [14] which failed to find significant stimulus effects on dual-axis accelerometry. However, the two studies differed in a number of key areas. First, they employed different segmentation schemes. Whereas the current study employed a segmentation algorithm based on sequential fuzzy c-means partitioning, our previous study utilized a moving window algorithm. This study also included manually segmented swallows, unlike the previous study. In fact, the differences in segmentation are reflected in swallow duration. The mean swallow duration reported in the previous study was 0.9577s, whereas the mean durations shown in Tables 1 and 2 are much longer. Second, although both studies analyzed identical signals acquired from the same participants, the actual swallows included in the statistical tests were different. This is largely due to the fact that the different segmentation algorithms led to the inclusion of different swallows during the visual verification step. Third, our previous study did not conduct separate statistical tests for the discrete and sequential tasks. This could have increased variability in feature values, which in turn could have masked stimulus effects. Fourth, most of the features in Tables 1 and 2 were not considered in our previous study. Fifth, the two studies pre-processed the raw signals in different ways. Not only were the wavelet denoising schemes slightly different, but the FIR filters used in this study for canceling the effects of the data acquisition system were not utilized in the previous study.

It should be noted that the present study investigated only 4 stimulus types. Considering that there are many variables that characterize a stimulus, the 4 stimuli represent only a small subset of possible bolus types, although they are important stimuli from a clinical perspective. Therefore, the reader is cautioned against widely generalizing these results to other stimuli.

In such instructed swallowing tasks as the ones utilized in this study, it has been shown that sip size variability is small across different sips [46,47]. Therefore, it is assumed that bolus volume was not a major factor in this study. Although it is important to acknowledge that bolus volume was not controlled in this study, the reader should note that volume can be viewed as one of many variables that characterize stimuli. Any volume effects associated with intake of different stimuli, if present, are also part of the shown stimulus effects. The interpretation of the stimulus effects in this section was primarily based on viscosity because viscosity is intuitively the most obvious difference among the four stimulus types. The reader is advised that the objective of this study was to investigate stimulus effects, not just viscosity effects.

The results of this study only indicate that certain signal characteristics of dual-axis accelerometry are stimulus dependent. A limitation here is that we do not necessarily know what this dependence implies for the clinical capabilities of dual-axis accelerometry. In fact, the features examined in this study may not have meaningful clinical value. Dual-axis accelerometry is a new technique that is still under investigation, and its precise utility in clinical dysphagia assessment and rehabilitation is yet to be uncovered.

## Conclusions

In this article, we analyzed the effects of four different liquid stimuli on dual-axis swallowing accelerometry signals. The analysis employed 30 time and time-frequency domain features. Most of the features did not exhibit statistically significant differences across the stimuli. However, entropy rate, L-Z complexity, and swallow duration showed a dependence on stimulus type. Specifically, it was shown that increasing viscosity induced longer swallows with decreasing L-Z complexity and greater entropy rate, which agree with swallowing physiology. Therefore, we conclude that stimulus effects should be considered in future swallowing accelerometry studies.

## Competing interests

The authors declare that they have no competing interests.

## Authors' contributions

JL carried out the data collection, conducted all mathematical computations and statistical tests, and wrote the entire manuscript. ES participated in the selection of the features employed in the study, carried out swallow segmentation, and programmed some of the feature extraction routines. CS designed the data collection protocol, suggested the main objective of the manuscript, provided supervision, and critically revised the manuscript. TC provided supervision and critically revised the manuscript. All authors read and approved the final manuscript.
